# Gastric Cancer in the Countryside or in the City: Does the Prognosis Change? An Analysis from the German States of Brandenburg and Berlin

**DOI:** 10.3390/curroncol32040228

**Published:** 2025-04-14

**Authors:** Andreas Loew, Anne von Ruesten, Constanze Schneider, René Mantke, Karsten H. Weylandt, Stephan Gretschel

**Affiliations:** 1 Medical Clinic B, University Hospital Ruppin-Brandenburg (UKRB), Brandenburg Medical School, 16816 Neuruppin, Germany; karsten.weylandt@mhb-fontane.de; 2 Clinical-Epidemiological Cancer Registry Brandenburg-Berlin (KKRBB), 03044 Cottbus, Germanyconstanze.schneider@kkrbb.de (C.S.); 3 Department of General and Visceral Surgery, Brandenburg University Hospital, Brandenburg Medical School, 14770 Brandenburg an der Havel, Germany; rene.mantke@mhb-fontane.de; 4 Faculty of Health Science Brandenburg, Joint Faculty of the Brandenburg University of Technology Cottbus-Senftenberg, the Brandenburg Medical School Theodor Fontane and the University of Potsdam, 14469 Potsdam, Germany; stephan.gretschel@mhb-fontane.de; 5 Department of General, Visceral, Thoracic and Vascular Surgery, University Hospital Ruppin-Brandenburg (UKRB), Brandenburg Medical School, 16816 Neuruppin, Germany

**Keywords:** gastric carcinoma, adenocarcinoma, epidemiology, real-world care, rural-urban comparison

## Abstract

**Background:** Medical care structures differ between urban and rural areas. Clinical cancer registries can depict real-world care through detailed data analysis, identifying potential regional disparities and contributing to improvements in healthcare. **Methods:** Data from the Brandenburg–Berlin Clinical-Epidemiological Cancer Registry for the years of diagnosis 2017–2022 were analyzed to assess the epidemiology and real-world care of gastric cancer, including cardia. Brandenburg was compared to Berlin regarding perioperative treatment regimens. The resulting survival benefits were assessed using Kaplan–Meier and Cox regression models. **Results:** For the years of diagnosis 2017 to 2022, 5805 cases of gastric carcinoma were documented in the cancer registry. Survival data showed no significant differences between Berlin and Brandenburg. Preoperative therapy for cT3/cT4N0 tumors was significantly more common in Berlin (72%) than in Brandenburg (68%). Perioperative therapy was associated with a survival benefit for stages T3-/T4N+ but not for stages T1N+ or T2. The lower proportion of pre-treated T3/T4N+ patients in rural Brandenburg did not result in a significant survival difference. **Conclusions:** The data provide a comprehensive representation of the current state of gastric cancer care in these two regions. Gastric cancer treatment outcomes, in terms of survival, are comparable between the rural region of Brandenburg and the urban center of Berlin.

## 1. Introduction

Gastric carcinoma is the fifth most common cancer worldwide and the fifth leading cause of cancer-related mortality [1]. The age-adjusted incidence for men is 7.1 in Western Europe and 16.2 in Eastern Europe, while for women it is 3.6 and 7.7, respectively (GLOBACAN 2022). In Germany, incidence rates have been declining for both genders since the early 2000s, though this trend is slowing. In 2020, the age-standardized incidence rate (ESR) was 13.8 for men and 6.7 for women [2]. Age-adjusted mortality rates are also declining in Germany and other countries [3].

Major risk factors include advanced age, *Helicobacter pylori* infection, reflux disease, and lifestyle habits. Treatment primarily depends on disease spread, but the biological characteristics of this molecularly and phenotypically heterogeneous disease are increasingly influencing systemic therapy. Perioperative systemic therapy has become standard for locally advanced gastric carcinoma due to its proven survival benefit [4,5], now extending to earlier stages (Ib) [6,7].

Systemic therapy for metastatic stages increasingly includes targeted therapies (anti-HER2, anti-Claudin-1), anti-angiogenic agents (anti-VEGF), and immunotherapies (anti-PD-1). Previous analyses have shown higher gastric cancer mortality rates in rural compared to urban populations [8], though these data are from the United States, where healthcare access is more variable.

Although Germany has a nationwide, uniform healthcare system, known for its unrestricted health care provision and patient-orientation, regional disparities persist in the structure and density of medical care across both outpatient and inpatient sectors (Source: Haschka RE, Schley K, Herwartz H: Provision of health care services and regional diversity in Germany: insights from a Bayesian health frontier analysis with spatial dependencies. *Eur. J. Health Econ.* 2020, 21:55–71). This study aims to compare treatment realities and outcomes between rural and metropolitan areas within Germany’s universal healthcare system, specifically focusing on Brandenburg (a predominantly rural state) and Berlin (Germany’s largest city).

The federal state of Brandenburg covers a surface area of 29,479 km^2^ and had 2.55 million inhabitants in 2023, while Berlin spans 891 km^2^ and had 3.66 million inhabitants (Source: Berlin-Brandenburg AfS: downloadstatistik-berlin-brandenburg.de 2023). In 2023, the physician density was 246 inhabitants per physician in Brandenburg, compared to 149 inhabitants per physician in Berlin. The number of hospital beds amounted to 14,970 in Brandenburg and 20,172 in Berlin (Source: Federal Statistical Office; German Medical Association, https://www.destatis.de accessed on 4 March 2025).

## 2. Materials and Methods

### 2.1. Data Collection

Data collection and analysis were conducted by the Brandenburg–Berlin Clinical-Epidemiological Cancer Registry (KKRBB) following a request from the Brandenburg Gastrointestinal Tumor Working Group. The quality and completeness of the cancer registry data is guaranteed by a reporting obligation by law. Furthermore, the financing of the cancer registries is currently subject to 43 funding criteria of statutory health insurance. Fulfilment of the funding criteria guarantees, among other things, sufficient completeness of the cancer diagnosis data.

Data were extracted for all first-diagnosed gastric carcinomas (ICD-10-GM: C16) from 2017 to 2022 (diagnosis years), including cardia carcinoma (C16.0), with the data status 1 June 2024. The data status reflects the date on which the data tables for this analysis were created.

### 2.2. Case Selection

For clinical evaluations of treatment (preoperative chemotherapy), only adenocarcinomas (ICD-O-3) were included. Other histological subtypes were excluded due to low case numbers. Clinical evaluations of preoperative chemotherapy were restricted to specific clinical tumor stages (cT3/cT4M0 or cT2 and cT1N+M0) based on the 2017 UICC TNM classification (8th edition). Data were analyzed based on the patient’s place of residence, though treatment could occur in other federal states.

### 2.3. Analyses and Statistical Methods

Crude and age-standardized incidence rates (ESR, based on the old European standard) were calculated to analyze temporal trends and facilitate international comparisons. Univariate linear regression models were used to assess trends in incidence rates over time.

Survival after cancer diagnosis was analyzed using univariate and multivariate Cox regression models. Absolute survival rates were calculated using the Kaplan–Meier method and compared using the log-rank test. Group comparisons were performed using the Chi-squared test. Statistical analyses were conducted using SPSS (Version 24) and R Studio (Version 4.3.0).

## 3. Results

### 3.1. Epidemiology

**Figure 1:**
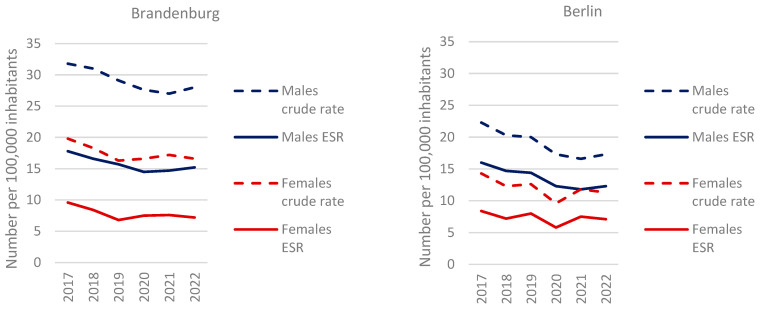
Crude new case rates, age-standardized new case rates (according to the old European standard, ESR). Comparison of women/men and Brandenburg/Berlin, diagnosis years 2017–2022.

For the years of diagnosis 2017 to 2022, 5805 new cases of gastric carcinoma were recorded, including 1632 (28%) cases with cardia involvement. The standardized incidence rate per 100,000 inhabitants (ESR) for men was 15.2 in Brandenburg and 12.3 in Berlin in 2022, marking a 24% higher incidence in the rural region. Incidence rates were 2.1 times higher in men than in women in Brandenburg (7.2) and 1.7 times higher in Berlin (7.1). Overall, incidence rates declined, with a significant linear trend for men (ESR: *p* = 0.034 for Brandenburg, *p* = 0.008 for Berlin) but not for women. The age distribution reflects the well-documented predominance of initial cases in older age groups, with a median age of 70.9 years for men and 74.1 years for women (Figure 1).

### 3.2. Survival Data

Men demonstrated significantly poorer survival compared to women in the overall cohort. This effect remained independent of age, place of residence, tumor location and clinical stage, documented preoperative chemotherapy, and ECOG performance status (multivariate hazard ratio: 1.09 [95% CI: 1.01–1.17], *p* = 0.028; see Table A1, Appendix A). Furthermore, the multivariate analysis identified age, clinical tumor stage, preoperative chemotherapy, and ECOG performance status as independent prognostic factors for overall survival. Notably, clinical staging information was available for approximately 70% of patients.

**Figure 2:**
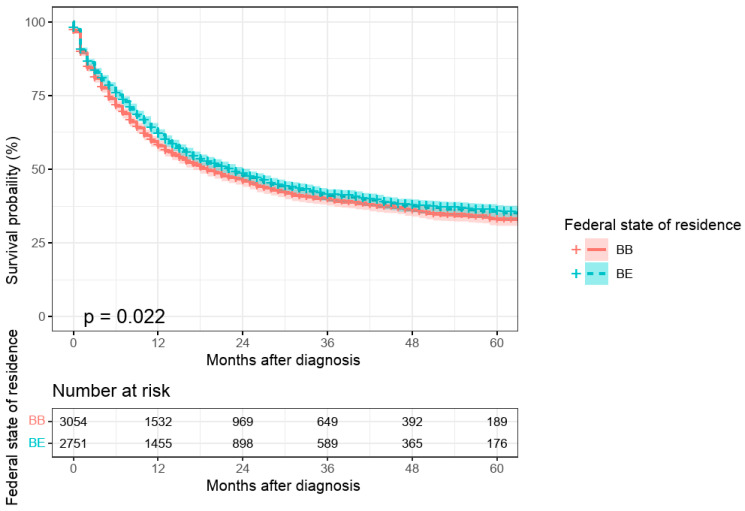
Absolute 5-year survival by place of residence in patients with gastric cancer, diagnosis years 2017–2022.

An initial univariate comparison between urban and rural areas indicated poorer survival for patients residing in Brandenburg compared to those in Berlin (*p* = 0.022; Figure 2). However, this effect was no longer significant in the multivariate model after adjusting for other risk factors (*p* = 0.722; see Table A1, Appendix A). Similarly, patients with cardiac carcinomas exhibited significantly worse survival than those with non-cardiac carcinomas in the univariate analysis (*p* = 0.038). However, this difference lost statistical significance in the multivariate model (*p* = 0.423; see Table A1, Appendix A).

### 3.3. Perioperative Therapy and Survival Outcomes

**Figure 3:**
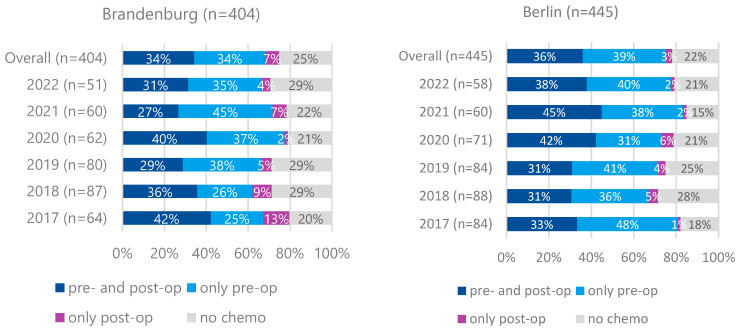
Proportion of pre- and postoperative chemotherapy for patients with gastric carcinoma who have undergone surgery, cT3 or cT4, M0, Brandenburg vs. Berlin, diagnosis years 2017–2022 (n = 849).

Preoperative therapy for cT3/cT4M0 tumors was less frequently administered in Brandenburg (68%) than in Berlin (75%) (*p* = 0.024). Preoperative and additional postoperative therapy rates were comparable between both regions (34% vs. 36%, *p* = 0.584). No increasing trend in preoperative therapy initiation was observed over time (Figure 3).

The FLOT regimen was the most commonly used protocol (81% in Brandenburg, 79% in Berlin), followed by FOLFOX (7% vs. 12%).

**Figure 4:**
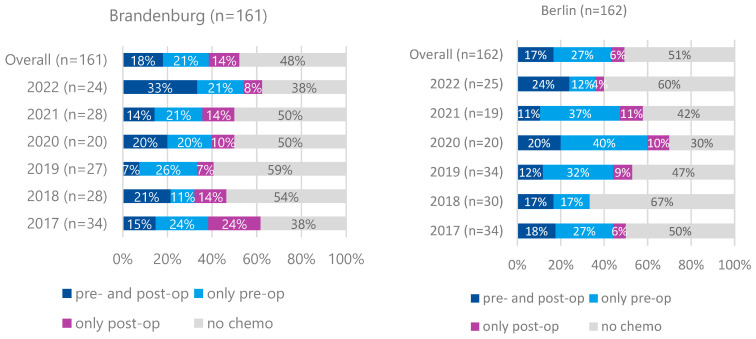
Proportion of pre- and postoperative chemotherapy for patients with gastric carcinoma who have undergone surgery, cT2 und cT1N+, M0, Brandenburg vs. Berlin, diagnosis years 2017–2022 (n = 323).

**Figure 5:**
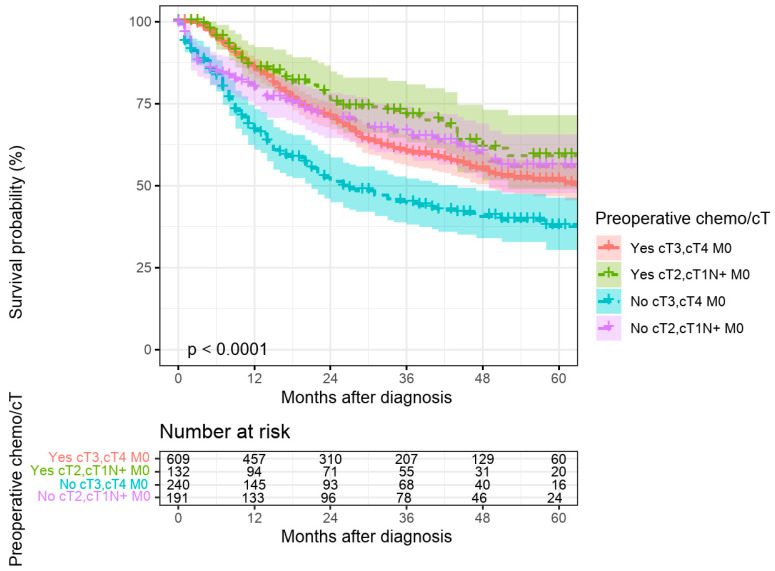
Absolute 5-year survival depending on preoperative chemotherapy separated by cTNM in patients with gastric carcinoma who have undergone surgery, place of residence Brandenburg and Berlin, diagnosis years 2017–2022.

For cT2 and cT1N+M0 cases, preoperative therapy rates were lower (39% in Brandenburg, 44% in Berlin) without significant differences between the regions (Figure 4). Preoperative therapy was associated with significantly improved 5-year survival for cT3/cT4M0 cases but not for cT2 or cT1N+M0 cases (Figure 5). The significant association of preoperative treatment with improved survival for the cT3, cT4, M0 cases was stable after multivariate adjustment (HR multivariate = 1.70 (1.33–2.17, *p* < 0.001, see Table 1). Furthermore, no association between federal state of residence and survival was observed among cT3, cT4, M0 as well as cT2, cT1N+, M0 cases (Table 1 and Table 2).

### 3.4. Patient Migration

The treatment of localized (non-metastatic) stages outside patients’ place of residence was observed in a cohort of 200 patients (35%) from Brandenburg, who underwent surgical treatment outside the region, with 80.5% of these surgeries performed in Berlin. Among these 200 cases, 63% also received preoperative chemotherapy in Berlin. Over the observation period, the proportion of patients seeking treatment outside their place of residence (Brandenburg) increased.

Subgroup analysis revealed no significant differences in the distribution of T categories, gender, or ECOG performance status between patients who underwent surgery outside Brandenburg and those treated surgically within the region. Furthermore, no differences were observed in absolute 5-year survival rates or surgical outcomes (local R).

However, patients who underwent surgery outside Brandenburg were significantly younger, had a higher rate of preoperative treatment (particularly neoadjuvant FLOT), and exhibited a higher prevalence of lymph node metastasis and cardiac carcinoma compared to those treated surgically within Brandenburg (Table A2).

## 4. Discussion

This study aimed to analyze data from the Brandenburg–Berlin clinical-epidemiological cancer registry to assess gastric cancer care in a metropolitan setting (Berlin) compared to a predominantly rural region (Brandenburg).

The standardized incidence rate of gastric cancer in men was markedly higher in Brandenburg than in Berlin and exceeded the national average for Germany [2]. The overall decline in incidence observed in both federal states aligns with international trends. In contrast, the USA has demonstrated stable incidence rates for men over the same period following a previous decline [9]. The higher incidence in men in Brandenburg may be linked to modifiable risk factors, such as smoking, alcohol consumption, environmental exposures, or an increased prevalence of *Helicobacter pylori* infection. However, our data do not allow for a direct confirmation of these factors [10]. The observed survival advantage of women over men is consistent with international findings [9,11,12].

A key limitation of this study is the incomplete reporting of clinical tumor stages to the cancer registry, with approximately 30% missing data. Improving the completeness of clinical staging is essential to enhance data reliability and allow for more precise comparisons.

Survival analyses revealed no significant differences between patients residing in Berlin and Brandenburg. This contrasts with findings from the SEER database in the USA, which indicated a survival disadvantage for patients from rural regions, particularly those with localized disease [13]. The difference may be attributed to Germany’s universal healthcare system, which ensures access to standardized oncologic care regardless of geographical location. Structural differences in healthcare delivery between urban and rural areas may still exist but appear not to impact overall survival significantly. Regarding treatment patterns, preoperative therapy was initiated in over two-thirds of patients with locally advanced gastric cancer (cT3/T4 M0), with a significantly higher rate in Berlin than in Brandenburg. However, only about half of the patients who received preoperative therapy also completed postoperative treatment. This aligns with clinical trial data: in the MAGIC study (perioperative ECF), 49.5% of patients completed postoperative therapy, while in the FLOT-4 study, completion rates were 61% for the FLOT arm and 50% for the ECF/ECX arm [14].

For patients with cT2 or cT1N+ tumors, the proportion receiving preoperative treatment was lower in both states, as expected. The German S3 guidelines (version 1.0 from 2012 and version 2 from 2019) provided a discretionary recommendation for perioperative treatment in these cases, permitting primary surgery as an alternative. Our survival data confirm the benefit of multimodal therapy for cT3/T4 N+ stages but not for cT1N+ or cT2 stages. The slightly lower proportion of pretreated patients in rural Brandenburg did not result in a significant survival disadvantage. We do not have data on economic factors or education level; however, the unrestricted healthcare provision in the German healthcare system ensures guideline-directed therapy, independent of income or education level.

As the German healthcare system allows free choice of providers, we observed patients residing in Brandenburg utilizing the medical infrastructure of the neighboring metropolis. In certain cases, decision-making was potentially influenced by the tumor location (cardia). A sensitivity analysis of this subgroup did not reveal significant differences in survival, but it did show a higher proportion of patients receiving preoperative chemotherapy, which we interpret, in summary, as a potential signal.

## 5. Conclusions

In Germany’s universal healthcare system, no significant differences in gastric cancer treatment outcomes were observed between the rural region of Brandenburg and the urban city of Berlin. These findings suggest that in a healthcare system with universal coverage, regional disparities in medical infrastructure do not necessarily translate into differences in patient survival. While registry reporting should be improved, the data reliably depict current care realities. Oncological providers should be encouraged to initiate perioperative systemic therapies for gastric cancer when indicated.

## Figures and Tables

**Table 1:** Survival according to preoperative chemotherapy and additional selected factors in surgically treated patients with gastric carcinoma (cT3, cT4, M0), residing in Brandenburg and Berlin, diagnosed between 2017 and 2022.

Factor	Category	N (%)	HR (Univariate)	HR (Multivariate)
Preoperative chemotherapy	Yes	609 (71.7)	-	-
	No	240 (28.3)	1.73 (1.39–2.16, *p* < 0.001)	1.70 (1.33–2.17, *p* < 0.001)
Sex	Women	274 (32.3)	-	-
	Men	575 (67.7)	1.00 (0.80–1.26, *p* = 0.987)	1.01 (0.80–1.28, *p* = 0.928)
Age	Mean (SD)	67.9 (11.6)	1.02 (1.01–1.03, *p* < 0.001)	1.02 (1.01–1.03, *p* = 0.002)
ECOG performance status	ECOG 0	320 (37.7)	-	-
	ECOG 1	191 (22.5)	0.86 (0.64–1.15, *p* = 0.304)	0.74 (0.55–1.01, *p* = 0.058)
	ECOG 2	63 (7.4)	1.46 (1.00–2.13, *p* = 0.050)	1.22 (0.82–1.79, *p* = 0.325)
	ECOG > 2	6 (0.7)	2.96 (1.09–8.03, *p* = 0.033)	2.62 (0.94–7.34, *p* = 0.067)
	Not available	269 (31.7)	0.99 (0.76–1.28, *p* = 0.929)	0.95 (0.73–1.23, *p* = 0.697)
cN category	N0	239 (28.2)	-	-
	N1	404 (47.6)	1.10 (0.84–1.43, *p* = 0.494)	1.22 (0.93–1.61, *p* = 0.146)
	N2	108 (12.7)	1.47 (1.04–2.08, *p* = 0.027)	1.67 (1.17–2.39, *p* = 0.005)
	N3	40 (4.7)	2.59 (1.68–3.99, *p* < 0.001)	2.81 (1.81–4.36, *p* < 0.001)
	NX	58 (6.8)	1.05 (0.65–1.71, *p* = 0.835)	1.05 (0.64–1.71, *p* = 0.841)
Place of residence	Berlin	445 (52.4)	-	-
	Brandenburg	404 (47.6)	1.07 (0.86–1.32, *p* = 0.533)	1.05 (0.84–1.31, *p* = 0.649)
Cardiac carcinoma	Yes	220 (25.9)	-	-
	No	629 (74.1)	0.96 (0.76–1.23, *p* = 0.773)	0.86 (0.66–1.12, *p* = 0.263)

**Table 2:** Survival according to preoperative chemotherapy and additional selected factors in surgically treated patients with gastric carcinoma (cT2, cT1N+, M0), residing in Brandenburg and Berlin, diagnosed between 2017 and 2022.

Factor	Category	N (%)	HR (Univariate)	HR (Multivariate)
Preoperative chemotherapy	Yes	132 (40.9)	-	-
	No	191 (59.1)	1.31 (0.88–1.96, *p* = 0.186)	1.24 (0.77–1.99, *p* = 0.374)
Sex	Women	129 (39.9)	-	-
	Men	194 (60.1)	1.24 (0.83–1.84, *p* = 0.288)	1.25 (0.82–1.90, *p* = 0.297)
Age	Mean (SD)	69.6 (12.2)	1.03 (1.01–1.05, *p* = 0.003)	1.03 (1.00–1.05, *p* = 0.015)
ECOG performance status	ECOG 0	122 (37.8)	-	-
	ECOG 1	67 (20.7)	1.94 (1.15–3.27, *p* = 0.012)	1.69 (1.00–2.88, *p* = 0.051)
	ECOG 2	15 (4.6)	1.59 (0.66–3.81, *p* = 0.302)	1.11 (0.44–2.77, *p* = 0.823)
	ECOG > 2	8 (2.5)	5.02 (1.94–12.98, *p* = 0.001)	3.70 (1.27–10.78, *p* = 0.017)
	Not available	111 (34.4)	1.27 (0.78–2.07, *p* = 0.333)	1.17 (0.71–1.92, *p* = 0.538)
cN category	N0	191 (59.1)	-	-
	N1	96 (29.7)	1.39 (0.92–2.12, *p* = 0.121)	1.57 (0.98–2.51, *p* = 0.058)
	N2	12 (3.7)	1.33 (0.48–3.67, *p* = 0.587)	1.21 (0.40–3.71, *p* = 0.736)
	N3	2 (0.6)	1.40 (0.19–10.10, *p* = 0.741)	1.39 (0.19–10.44, *p* = 0.748)
	NX	22 (6.8)	1.81 (0.86–3.81, *p* = 0.119)	1.54 (0.70–3.39, *p* = 0.280)
Place of residence	Berlin	162 (50.2)	-	-
	Brandenburg	161 (49.8)	0.96 (0.65–1.41, *p* = 0.822)	0.92 (0.62–1.38, *p* = 0.689)
Cardiac carcinoma	Yes	59 (18.3)	-	-
	No	264 (81.7)	0.84 (0.51–1.38, *p* = 0.491)	0.89 (0.52–1.51, *p* = 0.660)

## Data Availability

Data can be obtained from the Clinical-Epidemiological Cancer Registry Brandenburg–Berlin GmbH upon reasonable request.

## Appendix A

**Table A1:** Survival according to selected factors in patients with gastric carcinoma, residing in Brandenburg and Berlin, diagnosed between 2017 and 2022.

Factor	Category	N (%)	HR (Univariat)	HR (Multivariat)
Sex	Women	2199 (37.9)	-	-
	Men	3606 (62.1)	1.08 (1.01–1.17, *p* = 0.030)	1.09 (1.01–1.17, *p* = 0.028)
Age	Mean (SD)	70.5 (12.5)	1.02 (1.02–1.02, *p* < 0.001)	1.02 (1.01–1.02, *p* < 0.001)
Federal state of residence	Berlin	2751 (47.4)	-	-
	Brandenburg	3054 (52.6)	1.09 (1.01–1.16, *p* = 0.022)	1.01 (0.94–1.09, *p* = 0.722)
Cardiac carcinoma	Yes	1632 (28.1)	-	-
	No	4173 (71.9)	0.92 (0.85–1.00, *p* = 0.038)	1.03 (0.95–1.12, *p* = 0.423)
Clinical tumor stage	I	624 (10.7)	-	-
	II	625 (10.8)	1.21 (1.01–1.45, *p* = 0.036)	1.42 (1.19–1.70, *p* < 0.001)
	III	902 (15.5)	1.47 (1.25–1.73, *p* < 0.001)	1.91 (1.61–2.26, *p* < 0.001)
	IV	1875 (32.3)	3.94 (3.42–4.55, *p* < 0.001)	4.16 (3.60–4.81, *p* < 0.001)
	X	1779 (30.6)	1.41 (1.21–1.64, *p* < 0.001)	1.31 (1.13–1.52, *p* < 0.001)
Preoperative chemotherapy	Yes	1079 (18.6)	-	-
	No	4726 (81.4)	2.14 (1.93–2.38, *p* < 0.001)	1.76 (1.57–1.97, *p* < 0.001)
ECOG	ECOG 0	1538 (26.5)	-	-
	ECOG 1	1217 (21.0)	1.63 (1.47–1.82, *p* < 0.001)	1.31 (1.18–1.46, *p* < 0.001)
	ECOG 2	537 (9.3)	2.22 (1.95–2.53, *p* < 0.001)	1.70 (1.49–1.94, *p* < 0.001)
	ECOG > 2	258 (4.4)	3.94 (3.36–4.61, *p* < 0.001)	2.65 (2.25–3.13, *p* < 0.001)
	Not available	2255 (38.8)	1.47 (1.34–1.62, *p* < 0.001)	1.39 (1.26–1.54, *p* < 0.001)

**Table A2:** General characteristics of patients with (cT3, cT4 and cT2, cT1N+, M0) and residing in Brandenburg with surgery performed outside of Brandenburg.

Factor	Surgery Outside (n = 200)	Surgery in BB (n = 365)	*p*-Value
Age (years)			0.003
Mean (SD)	66.9 (11.1)	69.9 (11.2)	
Sex, n (%)			0.633
Female	67 (33.5%)	131 (35.9%)	
Male	133 (66.5%)	234 (64.1%)	
Preoperative chemotherapy, n (%)			<0.001
Yes	148 (74.0%)	189 (51.8%)	
No	52 (26.0%)	176 (48.2%)	
Clinical UICC stage, n (%)			<0.001
I	25 (12.5%)	75 (20.5%)	
II	55 (27.5%)	148 (40.5%)	
III	95 (47.5%)	124 (34.0%)	
IV	21 (10.5%)	13 (3.6%)	
X	4 (2.0%)	5 (1.4%)	
T-category, n (%)			0.246
T1	3 (1.5%)	3 (0.8%)	
T2	47 (23.5%)	108 (29.6%)	
T3	112 (56.0%)	202 (55.3%)	
T4	38 (19.0%)	52 (14.2%)	
cN-category, n (%)			<0.001
N0	56 (28.0%)	168 (46.0%)	
N1	105 (52.5%)	121 (33.2%)	
N2	18 (9.0%)	40 (11.0%)	
N3	11 (5.5%)	12 (3.3%)	
NX	10 (5.0%)	24 (6.6%)	
Cardia carcinoma, n (%)			<0.001
Yes	67 (33.5%)	46 (12.6%)	
No	133 (66.5%)	319 (87.4%)	
Local R classification, n (%)			0.714
R0	181 (90.5%)	327 (89.6%)	
R1	13 (6.5%)	31 (8.5%)	
R2	2 (1.0%)	2 (0.5%)	
RX	4 (2.0%)	5 (1.4%)	
